# Tiger Nut Milk’s Antiviral Properties against Enveloped and Non-Enveloped Viruses: Effect of Concentration and Adding Sugar

**DOI:** 10.3390/ijms241512018

**Published:** 2023-07-27

**Authors:** Alberto Tuñón-Molina, Alba Cano-Vicent, Ángel Serrano-Aroca

**Affiliations:** Biomaterials and Bioengineering Lab, Centro de Investigación Traslacional San Alberto Magno, Universidad Católica de Valencia San Vicente Mártir, 46001 Valencia, Spain; alberto.tunon@ucv.es (A.T.-M.); alba.cano@mail.ucv.es (A.C.-V.)

**Keywords:** horchata, tiger nut milk, sugar, bacteriophage phi 6, bacteriophage MS2, SARS-CoV-2

## Abstract

The global COVID-19 pandemic has warned scientists of the requirement to look for new antimicrobial compounds to prevent infection by this type of viral pathogen. Natural compounds are becoming a promising avenue of research thanks to their renewable, biodegradable, and non-toxic properties. In this work, tiger nut milk’s (TNM) antiviral properties, with and without sugar, were studied against enveloped and non-enveloped viruses. The antiviral properties of TNM were evaluated at different concentrations. The antiviral tests showed that TNM is antiviral against the enveloped bacteriophage phi 6, which is commonly used as a surrogate for severe acute respiratory syndrome coronavirus 2 (SARS-CoV-2), although it did not have any antiviral effect against the non-enveloped bacteriophage MS2. We also found that adding sugar to this natural drink can improve its antiviral properties against enveloped viruses and render it antiviral against non-enveloped viruses like bacteriophage MS2. The antiviral activity of TNM depends on the TNM concentration. TNM is a natural bioproduct that could help to fight against viral infections and protect against a wide range of viral illnesses. These results confirm that the typical sweetened drink made from tiger nut extract and sugar (known as horchata in Spain) possesses broad-spectrum antiviral properties.

## 1. Introduction

The COVID-19 pandemic has emphasized the need to find new approaches to any new threats that might show up in the future. Scientists have focused their efforts on finding new antimicrobial compounds that can help in this aim, and certain natural compounds have been described as potent antimicrobial substances. For example, glycyrrhizin is an antiviral agent obtained from the *Glycyrrhiza uralensis* plant [1] that has been shown to possess considerable antiviral activity against viruses such as hepatitis C virus, porcine reproductive virus, respiratory syndrome virus, pseudorabies virus, porcine epidemic diarrhea virus, and even severe acute respiratory syndrome coronavirus 2 (SARS-CoV-2) [1,2,3,4]. Cranberry extracts have also been found to be potent antiviral agents against viruses such as SARS-CoV-2, bacteriophage MS2, murine norovirus (MNV), or feline calicivirus. Cranberry-based compounds have also been reported to have beneficial effects against other pathogens, such as methicillin-resistant *Staphylococcus aureus* or methicillin-resistant *Staphylococcus epidermidis* bacteria and *Candida albicans* fungi [5,6,7,8].

Horchata, or tiger nut milk (TNM), is a natural drink typical of Valencia, Spain, that can be obtained from the tiger nut tuber (*Cyperus esculentus Lativum*) [9], is a sweet milky-textured drink, popular in the summer for its refreshing properties and with a rapid beneficial action against intestinal bacteria [10]. It has also been associated with preventing heart attacks and thrombosis, improving blood circulation [11,12], and reducing the risk of colon cancer [13].

Some studies have shown that TNM mixed with green tea extract has antiviral activity against viruses such as hepatitis A and MNV [14]. Tiger nut extract has been tested against different bacteria such as *Shigella* sp., *Salmonella* sp., *S. aureus*, and *Escherichia coli* to determine its possible antibacterial activity with very promising results [15]. However, as far as we know, there are no previous studies on the antiviral properties of TNM.

Considering the above results, we hypothesized that TNM could be a promising natural drink with antiviral properties against enveloped and non-enveloped viruses. The tests carried out included the analysis of the effects of concentrating and adding sugar on its antiviral properties.

## 2. Results and Discussion

### 2.1. Antiviral Tests against phi 6 Bacteriophage

Bacteriophage phi 6 possesses a round-like shape and a lipid envelope similar to that of SARS-CoV-2, which renders it very useful to be used as a surrogate of these highly infectious pathogenic particles for biosafety reasons [16].

**Figure 1:**
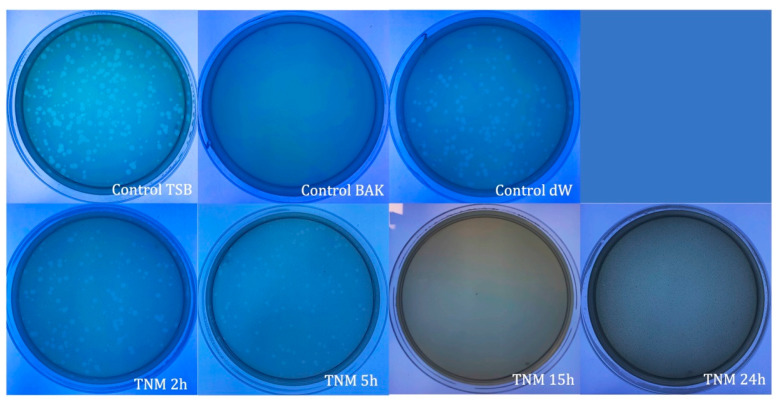
Loss of bacteriophage phi 6 viability measured by the double-layer method. Representative plate images of bacteriophages in contact with tryptic soy broth (TSB) (control TSB), antiviral benzalkonium chloride (BAK) (control BAK), and distilled water (control dW) for 24 h of viral contact. Plate images of bacteriophages in contact with tiger nut milk (TNM) for 2 h (TNM 2h), 5 h (TNM 5h), 15 h (TNM 15h), and 24 h (TNM 24h). After 15 h of contact, a complete inactivation of the virus is observed.

The TNM antiviral results against bacteriophage phi 6 showed 100% viral inactivation after 15 h and 24 h of viral contact (Figure 1 and Table 1).

Water (control dW), the main ingredient of TNM, did not affect the viral activity at any time (2, 5, 15, and 24 h), with no statistical difference from the control TSB. As expected, control BAK showed strong viral inactivation (100%) at all control times (2, 5, 15, and 24 h). Representative plate images and values of control TSB, control BAK, and control dW for 24 h of viral contact are shown in Figure 1 and Table 1, respectively.

**Figure 2:**
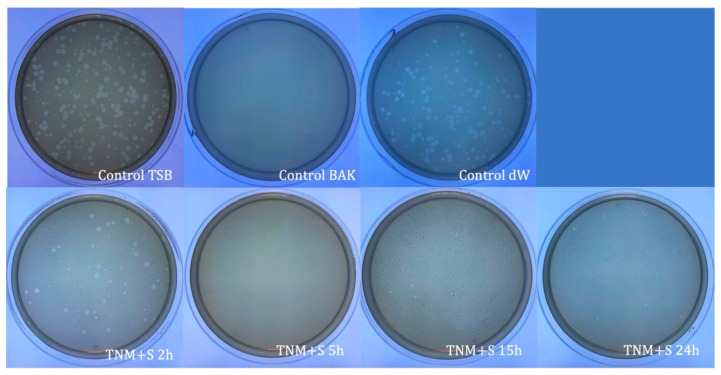
Loss of bacteriophage phi 6 viability measured by the double-layer method. Representative plate images of bacteriophages in contact with tryptic soy broth (TSB) (control TSB), antiviral benzalkonium chloride (BAK) (control BAK), and distilled water (control dW) for 24 h of viral contact. Plate images of bacteriophages in contact with tiger nut milk (TNM) with sugar for 2 h (TNM+S 2h), 5 h (TNM+S 5h), 15 h (TNM+S 15h), and 24 h (TNM+S 24h). After 5 h of contact, a complete inactivation of the viral activity is noticed.

TNM’s antiviral properties against bacteriophage phi 6 can be increased by adding 0.1 g/mL of sugar (Figure 2) when it can inactivate bacteriophage phi 6 (100% viral inactivation) in only 5 h (Table 2).

**Figure 3:**
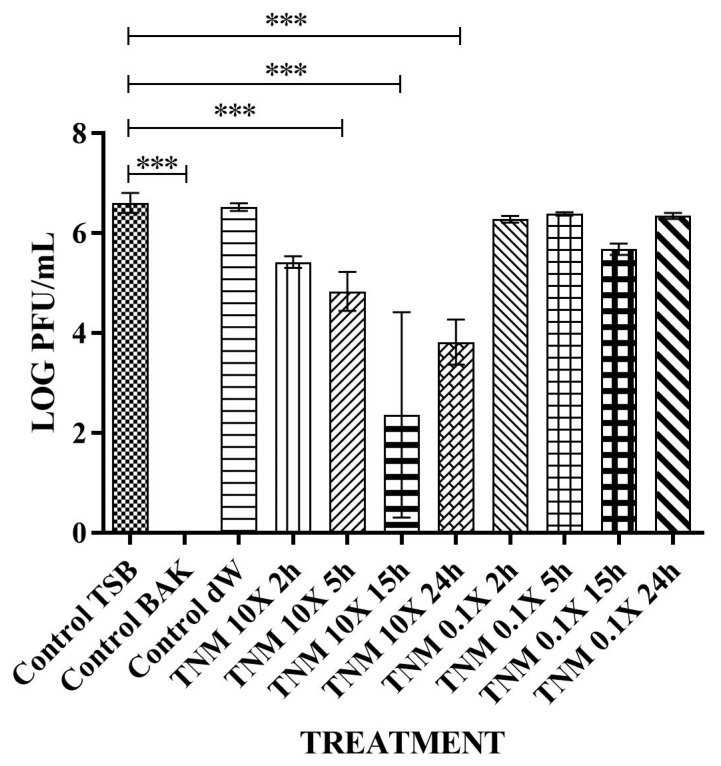
Reduction of phi 6 infection titers in the decadic logarithm of plaque-forming units per mL (LOG PFU/mL) measured by the double-layer method. Bacteriophages in contact with tiger nut milk (TNM) concentrated at 10X of initial concentration for 2 h (TNM 10X 2h), TNM concentrated at 10X of initial concentration for 5 h (TNM 10X 5h), TNM concentrated at 10X of initial concentration for 15 h (TNM 10X 15h), and TNM concentrated at 10X of initial concentration for 24 h (TNM 10X 24h). TNM concentrated at 0.1X of initial concentration for 2 h (TNM 0.1X 2h), bacteriophages in contact with TNM diluted at 0.1X of initial concentration for 5 h (TNM 0.1X 5h), TNM diluted at 0.1X of initial concentration for 15 h (TNM 0.1X 15h), and TNM diluted at 0.1X of initial concentration for 24 h (TNM 0.1X 24h). Representative values of bacteriophages in contact with tryptic soy broth (TSB) (control TSB), antiviral benzalkonium chloride (BAK) (control BAK), and distilled water (control dW) for 24 h of viral contact. Three independent antiviral tests were performed on two different days (*n* = 6). Significant differences with respect to control were determined by one-way ANOVA with Tukey’s correction for multiple comparisons: *** *p* < 0.001. A significant antiviral activity against bacteriophage phi 6 is achieved after only 5 h of contact with the 10X concentrated form of TNM. The 0.1X diluted form of TNM shows no antiviral properties after 24 h of contact.

After analyzing the results obtained with the commercial concentration, two different TNM concentrations were prepared at 10X and 0.1X of the initial concentration and tested against bacteriophage phi 6 (Figure 3). These concentrations were prepared to test if a 10X concentrated form of TNM could enhance its antiviral properties. The 0.1X diluted form assays were performed to find the minimal concentration that could inactivate the bacteriophage.

**Figure 4:**
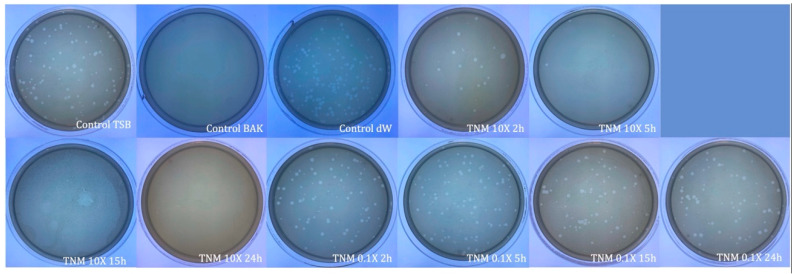
Loss of bacteriophage phi 6 viability measured by the double-layer method. Representative plate images of bacteriophages in contact with tryptic soy broth (TSB) (control TSB), antiviral benzalkonium chloride (BAK) (control BAK), and distilled water (control dW) for 24 h of viral contact. Plate images of bacteriophages in contact with concentrated tiger nut milk (TNM 10X) for 2 h (TNM 10X 2h); concentrated TNM 10X for 5 h (TNM 10X 5h); concentrated TNM 10X for 15 h (TNM 10X 15h); concentrated TNM 10X for 24 h (TNM 10X 24h); diluted TNM 0.1X for 2 h (TNM 0.1X 2h); diluted TNM 0.1X for 5 h (TNM 0.1X 5h); diluted TNM 0.1X for 15 h (TNM 0.1X 15h), and diluted TNM 0.1X for 24 h (TNM 0.1X 24h). Antiviral activity is shown after only 2 h of contact with a 10X concentrated form of TNM, increasing this activity as time of contact increases, too. No antiviral activity is observed with the 0.1X diluted form of TNM.

The results showed that 0.1X concentration was ineffective against this bacteriophage for any period of viral contact (Table 1 and Figure 4).

However, the TNM 10X concentration showed a ~1 logarithm reduction after only 2 h of contact, indicating 87% bacteriophage phi 6 deactivation. This reduction was higher after prolonged hours of contact, inactivating almost 100% of the viral particles after 15 h of contact (Table 3 and Figure 4).

Up to 51 different compounds have been identified in a TNM infusion [17]. Furthermore, that study confirmed by computational analysis that TNM contains potential inhibitors of the SARS-CoV-2 M^pro^ and PL^pro^ enzymes. Thus, three compounds were found as candidates for M^pro^ inhibition: benzoic acid, 2-(ethylthio)-ethyl ester, l-Leucine-*N*-isobutoxycarbonyl-*N*-methyl-heptyl, isorhamnetin, and for PL^pro^: isorhamnetin-3-O-(6-Orhamnosyl-galactoside), dihydroxy-methoxyflavanone and dihydroxyphenyl)-5-hydroxy-4-oxochromen-7-yl]oxy-3,4,5-trihydroxyoxane-2-carboxylic acid.

Sugar derivatives have shown broad-spectrum antiviral activity [18,19,20]. Therefore, these results show that TNM has antiviral properties both with and without sugar against an enveloped biosafe virus model (bacteriophage phi 6) of highly pathogenic enveloped viruses such as SARS-CoV-2, Ebola, and influenza.

### 2.2. Antiviral Tests against Bacteriophage MS2

**Figure 5:**
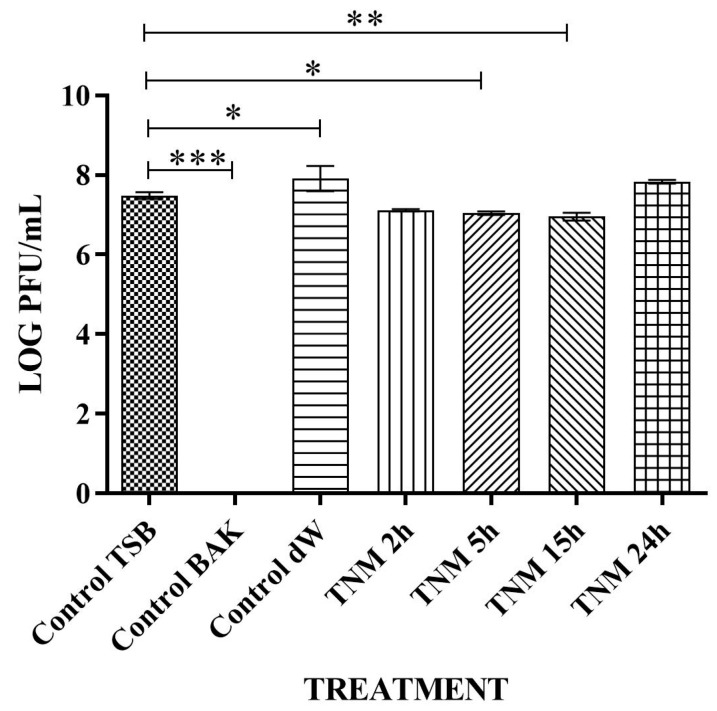
Reduction of infection titers of bacteriophage MS2 in the decadic logarithm of plaque-forming units per mL (LOG 10 PFU/mL) measured by the double-layer method. Bacteriophages in contact with tiger nut milk (TNM) at a commercial concentration (0.32 g/mL) for 2 h (TNM 2h), TNM for 5 h (TNM 5h), TNM for 15 h (TNM 15h), and TNM for 24 h (TNM 24h). Representative values of bacteriophages in contact with tryptic soy broth (TSB) (control TSB), antiviral benzalkonium chloride (BAK) (control BAK), and distilled water (control dW) for 24 h of viral contact. Three independent antiviral tests were performed on two different days (*n* = 6). Significant differences with respect to control were determined by one-way ANOVA with Tukey’s correction for multiple comparisons: * *p* < 0.05; ** *p* < 0.01; *** *p* < 0.001. No significant antiviral activity is observed after 24 h against bacteriophage MS2 with TNM at commercial concentration.

Antiviral tests with TNM at a commercial concentration (0.32 g/mL) against MS2 did not show any significant reduction of viral activity (Figure 5).

**Figure 6:**
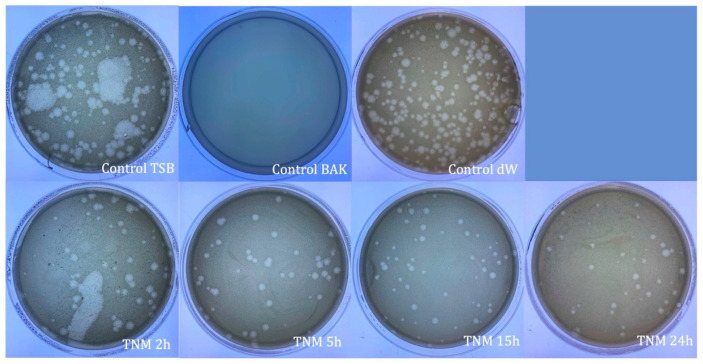
Loss of MS2 bacteriophage viability measured by the double-layer method. Representative plate images of bacteriophages in contact with tryptic soy broth (TSB) (control TSB), antiviral benzalkonium chloride (BAK) (control BAK), and distilled water (control dW) for 24 h of viral contact. Plate images of bacteriophages in contact with tiger nut milk (TNM) for 2 h (TNM 2h), TNM for 5 h (TNM 5h), TNM for 15 h (TNM 15h), and TNM for 24 h (TNM 24h).

No reduction of viral activity was evident after this time of viral contact (Table 4 and Figure 6).

**Figure 7:**
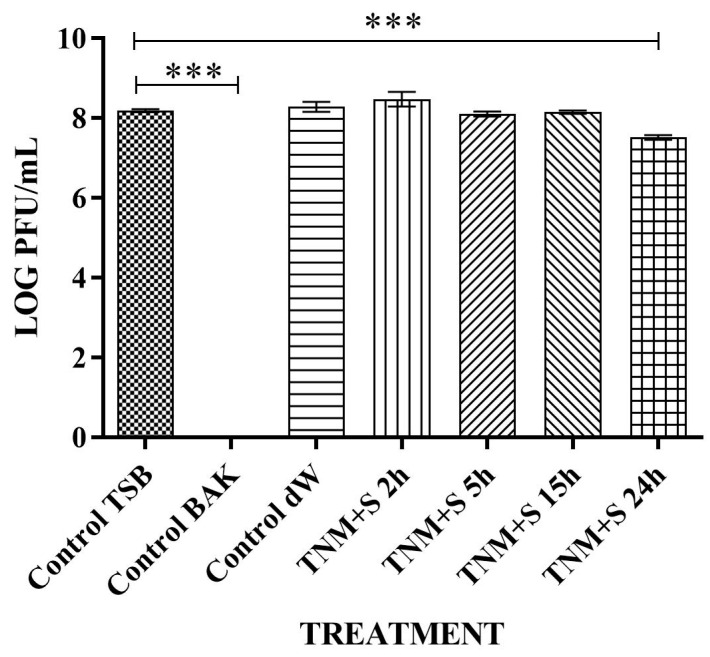
Reduction of infection titers of bacteriophage MS2 in the decadic logarithm of plaque-forming units per mL (LOG 10 PFU/mL) measured by the double-layer method. Representative values of bacteriophages in contact with tryptic soy broth (TSB) (control TSB), antiviral benzalkonium chloride (BAK) (control BAK), and distilled water (control dW) for 24 h of viral contact. MS2 bacteriophages in contact with tiger nut milk (TNM) with sugar for 2 h (TNM+S 2h), TNM with sugar for 5 h (TNM+S 5h), TNM with sugar for 15 h (TNM+S 15h), and TNM with sugar for 24 h (TNM+S 24h). Three independent antiviral tests were performed on two different days (*n* = 6). Significant differences with respect to control were determined by one-way ANOVA with Tukey’s correction for multiple comparisons: *** *p* < 0.001. A significant reduction of viral activity is observed after 24 h against bacteriophage MS2 with TNM with sugar.

However, when 0.1 g/mL of sugar was added to TNM, a statistically significant reduction of viral activity (~1 logarithm) was found after 24 h of contact (Figure 7).

**Figure 8:**
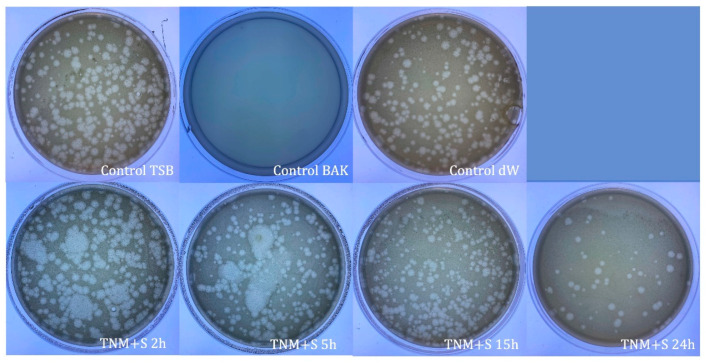
Loss of bacteriophage MS2 viability measured by the double-layer method. Representative plate images of bacteriophages in contact with tryptic soy broth (TSB) (control TSB), antiviral benzalkonium chloride (BAK) (control BAK), and distilled water (control dW) for 24 h of viral contact. Plate images of bacteriophages in contact with tiger nut milk (TNM) with sugar for 2 h (TNM+S 2h), TNM with sugar for 5 h (TNM+S 5h), TNM with sugar for 15 h (TNM+S 15h), and TNM with sugar for 24 h (TNM+S 24h). TNM with sugar shows antiviral properties against bacteriophage MS2 after 24 h of contact.

This viral inactivation at 24 h is 79% (Table 5 and Figure 8).

**Figure 9:**
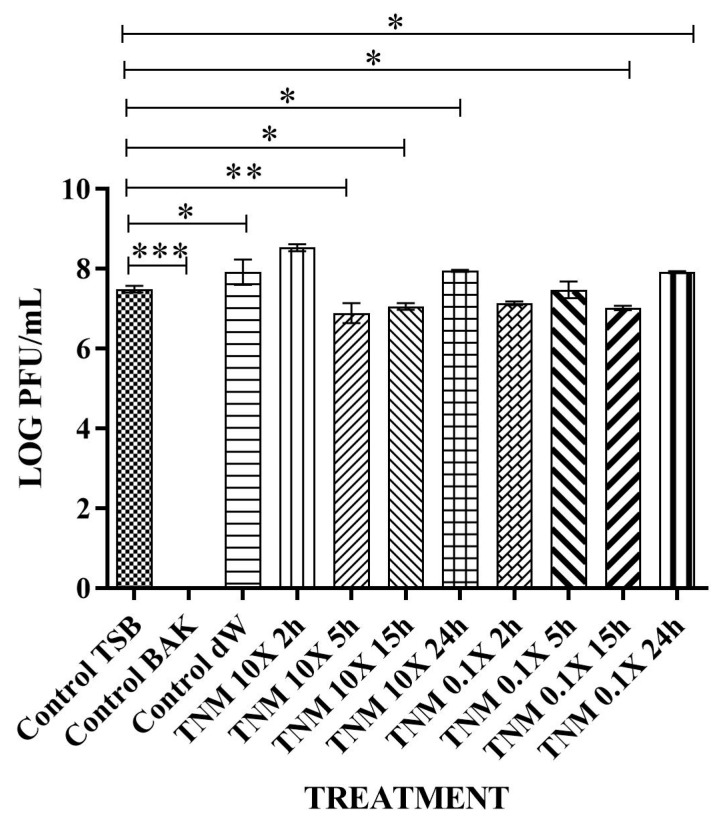
Reduction of infection titers of bacteriophage MS2 in the decadic logarithm of plaque-forming units per mL (LOG 10 PFU/mL) measured by the double-layer method. Representative values of bacteriophages in contact with tryptic soy broth (TSB) (control TSB), antiviral benzalkonium chloride (BAK) (control BAK), and distilled water (control dW) for 24 h of viral contact. MS2 bacteriophages in contact with tiger nut milk (TNM) concentrated at 10X of initial concentration for 2 h (TNM 10X 2h), TNM concentrated at 10X of initial concentration for 5 h (TNM 10X 5h), TNM concentrated at 10X of initial concentration for 15 h (TNM 10X 15h), and TNM concentrated at 10X of initial concentration for 24 h (TNM 10X 24h). MS2 bacteriophages in contact with TNM diluted at 0.1X of initial concentration for 2 h (TNM 0.1X 2h), TNM diluted at 0.1X of initial concentration for 5 h (TNM 0.1X 5h), TNM diluted at 0.1X of initial concentration for 15 h (TNM 0.1X 15h), and TNM diluted at 0.1X of initial concentration for 24 h (TNM 0.1X 24h) were also tested. Three independent antiviral tests were performed on two different days (*n* = 6). Significant differences with respect to control were determined by one-way ANOVA with Tukey’s correction for multiple comparisons: *** *p* < 0.001; ** *p* < 0.01; * *p* < 0.05. The 10X concentrated form and 0.1X diluted form of TNM showed no antiviral effect against bacteriophage MS2 after 24 h of contact.

TNM at a 10X concentration or diluted to 0.1X of the initial concentration did not show any significant antiviral activity against bacteriophage MS2 (Figure 9).

**Figure 10:**
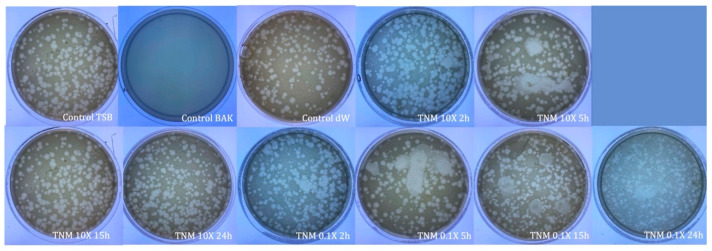
Loss of bacteriophage MS2 viability measured by the double-layer method. Representative plate images of bacteriophages in contact with tryptic soy broth (TSB) (control TSB), antiviral benzalkonium chloride (BAK) (control BAK), and distilled water (control dW) for 24 h of viral contact. Plates of bacteriophages in contact with concentrated tiger nut milk (TNM 10X) for 2 h (TNM 10X 2h); concentrated TNM 10X for 5 h (TNM 10X 5h); concentrated TNM 10X for 15 h (TNM 10X 15h); concentrated TNM 10X for 24 h (TNM 10X 24h); diluted TNM 0.1X for 2 h (TNM 0.1X 2h); diluted TNM 0.1X for 5 h (TNM 0.1X 5h); diluted TNM 0.1X for 15 h (TNM 0.1X 15h) and diluted TNM 0.1X for 24 h (TNM 0.1X 24h). No reduction of viral titers is observed when treated with the 10X concentrated form or 0.1X diluted form of TNM after 24 h of contact.

No reduction of viral activity was found even after 24 h of contact when testing the TNM concentrated (10X) or diluted (0.1X) forms (Table 6 and Figure 10).

Therefore, TNM without sugar has no antiviral activity against non-enveloped viruses such as bacteriophage MS2. These negative results can be attributed to the fact that non-enveloped viruses are more resistant to inactivation than enveloped viruses [21]. However, the addition of sugar to TNM provided antiviral activity against non-enveloped MS2, which is in good agreement with previous studies that have shown positive antiviral activity of different sugar derivatives against enveloped and non-enveloped viruses [18,19,20]. The typical sweetened drink made with tiger nut extracts and sugar can therefore be seen to possess broad-spectrum antiviral properties.

Antiviral properties could be explained because of the presence of flavonoids such as quercetin in the TNM [22]. Quercetin is a known flavonoid that possesses antibacterial properties against a large number of strains that can affect the gastrointestinal, respiratory, or urinary system. Moreover, there are also viruses that can be affected by flavonoids, such as adenovirus or the herpes simplex virus. The anti-replicative and anti-infective characteristics of flavonoids can contribute to their antiviral properties [23,24,25]. In a study carried out with onions, quercetin content increased to temperatures of 120 °C and decreased at 150 °C, demonstrating that degradation of this compound could occur in very specific and non-natural conditions [26].

## 3. Materials and Methods

### 3.1. Materials

Commercial TNM containing tiger nut extract and water only was provided by the Horchatería Rin, Alboraya, Spain.

To produce concentrated and dilute TNM, 200 mL of commercial TNM were introduced into a spherical flask and placed in an R-210 rotary evaporator (BÜCHI Labortechnik AG, Flawil, Switzerland) for 48 h at 30 °C to finally provide 64.78 g of dried TNM, i.e., commercial TNM has a concentration of 0.32 g of tiger nut extract per mL of water. Concentrated TNM (TNM 10X) was prepared by weighing 3.2 g of dried TNM and diluting it in 1 mL of distilled water and diluted TNM (TNM 0.1X) by weighing 0.032 g of dried TNM and diluting it in 1 mL of distilled water.

Sweet TNM (TNM + S) was prepared by adding the typical amount of 0.1 g of sugar (AB Azucarera Iberia, Madrid, Spain) per 1 mL of TNM.

### 3.2. Antiviral Tests against phi 6 Bacteriophage

In these antiviral tests, *Pseudomonas syringae* (DSM 21482) was infected by the phi 6 bacteriophage, used as a biosafe viral model of SARS-CoV-2 [16]. This Gram-negative bacterium was obtained from the Leibniz Institute DSMZ–German Collection of Microorganisms and cell cultures GmbH (Braunschweig, Germany). The bacterium was first grown in compact tryptic soy agar (TSA, Scharlab, Barcelona, Spain) and later in liquid tryptic soy broth (TSB, Scharlab, Barcelona, Spain) at a velocity of 240 rpm at 25 °C. The Leibniz Institute’s stipulations were followed to disseminate the phi 6 (DSM 21518). Its antiviral activity was tested by combining a mixture of 50 μL of TSB with bacteriophages in an Eppendorf tube at a titer of about 1 × 10^6^ plaque-forming units per mL (PFU/mL) and 50 μL of TNM 1X, TNM 0.1X, TNM + S 1X, or TNM 10X and incubated for different times (2 h, 5 h, 15 h, and 24 h) at 25 °C [27]. A 15 mL falcon tube was employed to set down 50 μL of each mixture with 10 mL TSB to be vortexed for 1 min at room temperature (24 ± 1 °C). Bacteriophage titration was carried out by serial dilutions of each falcon tube sample, while 100 μL of each bacteriophage dilution was mixed with 100 μL of the *P. syringae* at OD_600nm_ = 0.5. Bacteriophage phi 6 infection capability was studied by the double-layer method [28], in which 4 mL of top agar (TSB + 0.75% bacteriological agar, Scharlab) and 5 mM calcium chloride (Sigma-Aldrich, St. Louis, MO, USA) were added to the bacteriophage dispersion combined with the bacteria and then decanted onto TSA plates for incubation for 18–24 h in a thermostatic oven at 25 °C. Bacteriophage titers in PFU/mL of every assay were compared with the control (control TSB), which consisted of 50 μL of bacteriophage dilution in 10 mL of TSB added directly to the bacterial culture without any contact with TNM. Moreover, a separate control with distilled water (control dW) was performed to guarantee that the distilled water did not interfere with the bacteriophage or the infection procedure. For this control dW, 50 μL of the bacteriophage phi 6 solution at a concentration of 1 × 10^6^ PFU/mL was mixed with 50 μL of distilled water. Positive control with benzalkonium chloride (control BAK) was also tested to ensure the bacteriophage phi 6 was deactivated before contact with a known antiviral material [29,30]. The antiviral properties of the dilute TNM were determined at 2 h, 5 h, 15 h, and 24 h of exposure to the bacteriophage phi 6. After the assay, plates were placed over a white light transilluminator and were red visually, counting PFU one by one. Pictures were taken with an iPhone XR camera. It was verified that vortexing did not modify the phi 6 bacteriophage infection capacity and that the remaining disinfectants in the titrated samples did not intervene with titration. Three separate antiviral assays were carried out on two different days (*n* = 6) to ensure reproducibility.

### 3.3. Antiviral Tests against MS2 Bacteriophage

*E. coli* (DSM 5695) was infected by the MS2 bacteriophage in the antiviral tests. The bacterium was grown in solid tryptic soy agar (TSA, Scharlab, Barcelona, Spain) and later in liquid tryptic soy broth (TSB, Scharlab, Barcelona, Spain) at a speed of 240 rpm at 37 °C in an orbital shaker following the Leibniz Institute’s requirements. The antiviral test was performed by combining a diffusion of 50 μL of TSB with bacteriophages at a concentration of roughly 1 × 10^6^ plaque-forming units per mL (PFU/mL) with 50 μL of TNM dilution in an Eppendorf tube and incubated for different times (2 h, 5 h, 15 h, and 24 h) at 37 °C [27]. A 15 mL falcon tube was used to lay down 50 μL of each bacteriophage solution with 10 mL TSB to be vortexed for 1 min at ambient temperature (24 ± 1 °C). The bacteriophage solution was titrated by serial dilutions from each falcon tube sample, and 100 μL of a single bacteriophage dilution was homogenized with 100 μL of the bacterial host at OD_600nm_ = 0.13. The infectivity of the MS2 was studied by the double-layer method [28]: 4 mL of top agar (TSB + 0.75% bacteriological agar, Scharlab) and 5 mM of calcium chloride (Sigma-Aldrich, St. Louis, MO, USA) were joined to the bacteriophage dispersion, homogenized with the bacteria, and then drawn off on TSA plates for incubation in a thermostatic oven at 37 °C. Bacteriophage titers in PFU/mL of single samples were compared with the control (control TSB), prepared with 50 μL of bacteriophage dispersion in 10 mL of TSB joined to the bacterial culture without contact with any type of TNM dilution and a distilled water control (control dW), consisting of 50 μL of MS2 dispersed at a concentration of 1 × 10^6^ PFU/mL mixed with 50 μL of distilled water. Positive control with BAK was tested to guarantee that the bacteriophage phi 6 was deactivated when treated with a well-known antiviral material. The diluted TNM antiviral activity was determined at 2 h, 5 h, 15 h, and 24 h with MS2. After the assay, plates were placed over a white light transilluminator and were read visually, counting PFU one by one. Pictures were taken with an iPhone XR camera. The vortexing procedure was ensured not to influence the MS2 infectivity and that the remaining disinfectants in the titrated samples did not interfere with the titration method. Three separate antiviral assays were carried out on two different days (*n* = 6) to guarantee reproducibility.

### 3.4. Statistical Analysis

Significant differences with respect to control were determined by one-way ANOVA with Tukey’s correction for multiple comparisons using GraphPad Prism 6 software: * *p* < 0.05; ** *p* < 0.01; *** *p* < 0.001. Three independent antiviral tests were performed on two different days (*n* = 6).

## 4. Conclusions

The results reported here demonstrate TNM’s capacity to inactivate enveloped viruses against a surrogate of SARS-CoV-2, showing that TNM can be added to the list of natural products with antiviral effects in common with other natural compounds such as cranberry extracts or licorice. We have evaluated the antiviral effect of TNM concentration and adding sugar to TNM. Thus, sweet TNM showed antiviral properties against both enveloped and non-enveloped viruses, and the antiviral activity of TNM decreases with decreasing TNM concentration. These results thus provide valuable information on TNM’s antiviral properties with and without sugar against a broad spectrum of infectious viruses. However, in this study, only bacteriophage phi 6 and MS2 were used as representative viruses of enveloped and non-enveloped pathogens, respectively. Therefore, highly pathogenic viruses such as SARS-CoV-2, Ebola, influenza, and non-enveloped ones should be tested against TNM in order to confirm these excellent results in the future.

## Figures and Tables

**Table 1:** Antiviral results of tiger nut milk (TNM) at a commercial concentration (0.32 g/mL) against the phi 6 bacteriophage after 2, 5, 15, and 24 h of contact in plaque-forming units per mL (PFU/mL). Representative values of bacteriophages in contact with tryptic soy broth (TSB) (control TSB), antiviral benzalkonium chloride (BAK) (control BAK), and distilled water (control dW) for 24 h of viral contact.

SAMPLE	VIRUS CONCENTRATION (PFU/mL)	Log10 REDUCTION	VIRAL INACTIVATION (%)
control TSB	4.96 × 10^6^ ± 4.53 × 10^5^	-	-
control BAK	0.00 ± 0.00	~7	100
control dW	3.11 × 10^6^ ± 3.72 × 10^5^	~0	~0
TNM 2h	3.01 × 10^6^ ± 6.52 × 10^5^	~0	~0
TNM 5h	3.21 × 10^6^ ± 1.97 × 10^5^	~0	~0
TNM 15h	0.00 ± 0.00	~7	100
TNM 24h	0.00 ± 0.00	~7	10

**Table 2:** Antiviral effects of tiger nut milk (TNM) at a commercial concentration (0.32 g/mL) with 0.1 g/mL of sugar added against bacteriophage phi 6 after 2, 5, 15, and 24 h in plaque-forming units per mL (PFU/mL). Representative values of bacteriophages in contact with tryptic soy broth (TSB) (control TSB), antiviral benzalkonium chloride (BAK) (control BAK), and distilled water (control dW) for 24 h of viral contact.

SAMPLE	VIRUS CONCENTRATION (PFU/mL)	Log10 REDUCTION	VIRAL INACTIVATION (%)
control TSB	2.37 × 10^6^ ± 5.33 × 10^5^	-	-
control BAK	0.00 ± 0.00	~6	100
control dW	3.10 × 10^6^ ± 8.77 × 10^5^	~0	~0
TNM+S 2h	1.57 × 10^6^ ± 1.22 × 10^5^	~0	~0
TNM+S 5h	0.00 ± 0.00	~6	100
TNM+S 15h	0.00 ± 0.00	~6	100
TNM+S 24h	0.00 ± 0.00	~6	100

**Table 3:** Antiviral results of concentrated tiger nut milk (TNM 10X) and diluted TNM 0.1X forms against bacteriophage phi 6 after different times (2, 5, 15, and 24 h) of viral contact in plaque-forming units per mL (PFU/mL). Representative values of bacteriophages in contact with tryptic soy broth (TSB) (control TSB), antiviral benzalkonium chloride (BAK) (control BAK), and distilled water (control dW) for 24 h of viral contact.

SAMPLE	VIRUS CONCENTRATION (PFU/mL)	Log10 REDUCTION	VIRAL INACTIVATION (%)
control TSB	2.04 × 10^6^ ± 2.83 × 10^5^	-	-
control BAK	0.00 ± 0.00	~6	100
control dW	3.43 × 10^6^ ± 1.84 × 10^5^	~0	~0
TNM 10X 2h	2.69 × 10^5^ ± 7.26 × 10^4^	~1	~87
TNM 10X 5h	9.13 × 10^4^ ± 8.72 × 10^4^	~2	~96
TNM 10X 15h	2.67 × 10^3^ ± 3.06 × 10^3^	~3	~100
TNM 10X 24h	8.67 × 10^3^ ± 6.11 × 10^3^	~3	~100
TNM 0.1X 2h	1.92 × 10^6^ ± 2.88 × 10^5^	~0	~0
TNM 0.1X 5h	2.43 × 10^6^ ± 1.67 × 10^5^	~0	~0
TNM 0.1X 15h	4.87 × 10^5^ ± 1.30 × 10^5^	~0.5	~76
TNM 0.1X 24h	2.21 × 10^6^ ± 3.03 × 10^5^	~0	~0

**Table 4:** Antiviral results of tiger nut milk (TNM) at a commercial concentration (0.32 g/mL) against MS2 bacteriophage after 2, 5, 15, and 24 h of viral contact in plaque-forming units per mL (PFU/mL). Representative values of bacteriophages in contact with tryptic soy broth (TSB) (control TSB), antiviral benzalkonium chloride (BAK) (control BAK), and distilled water (control dW) 24 h of viral contact.

SAMPLE	VIRUS CONCENTRATION (PFU/mL)	Log10 REDUCTION	VIRAL INACTIVATION (%)
control TSB	4.40 × 10^7^ ± 5.66 × 10^6^	-	-
control BAK	0.00 ± 0.00	~8	100
control dW	2.17 × 10^7^ ± 6.23 × 10^6^	~0	~0
TNM 2h	1.31 × 10^7^ ± 9.45 × 10^5^	~0	~0
TNM 5h	1.11 × 10^7^ ± 1.21 × 10^6^	~0	~0
TNM 15h	9.27 × 10^6^ ± 2.21 × 10^6^	~0	~0
TNM 24h	6.80 × 10^7^ ± 6.93 × 10^6^	~0	~0

**Table 5:** Antiviral results of tiger nut milk (TNM) with 0.1 g/mL of sugar added against bacteriophage MS2 after 2, 5, 15, and 24 h of contact in plaque-forming units per mL (PFU/mL). Representative values of bacteriophages in contact with tryptic soy broth (TSB) (control TSB), antiviral benzalkonium chloride (BAK) (control BAK), and distilled water (control dW) for 24 h of viral contact.

SAMPLE	VIRUS CONCENTRATION (PFU/mL)	Log10 REDUCTION	VIRAL INACTIVATION (%)
control TSB	1.54 × 10^8^ ± 1.41 × 10^7^	-	-
control BAK	0.00 ± 0.00	~8	100
control dW	2.17 × 10^8^ ± 6.23 × 10^7^	~0	~0
TNM+S 2h	7.47 × 10^8^ ± 6.11 × 10^7^	~0	~0
TNM+S 5h	1.14 × 10^8^ ± 4.06 × 10^7^	~0	~0
TNM+S 15h	1.17 × 10^8^ ± 1.92 × 10^7^	~0	~0
TNM+S 24h	3.23 × 10^7^ ± 4.41 × 10^6^	~1	~79

**Table 6:** Antiviral results of concentrated tiger nut milk (TNM 10X) and diluted (TNM 0.1X) forms against bacteriophage MS2 after 2, 5, 15, and 24 h contact in plaque-forming units per mL (PFU/mL). Representative values of bacteriophages in contact with tryptic soy broth (TSB) (control TSB), antiviral benzalkonium chloride (BAK) (control BAK), and distilled water (control dW) for 24 h of viral contact.

SAMPLE	VIRUS CONCENTRATION (PFU/mL)	Log10 REDUCTION	VIRAL INACTIVATION (%)
control TSB	2.64 × 10^7^ ± 1.13 × 10^6^	-	-
control BAK	0.00 ± 0.00	~7	100
control dW	5.64 × 10^7^ ± 2.34 × 10^6^	~0	~0
TNM 10X 2h	1.44 × 10^7^ ± 2.31 × 10^6^	~0	~0
TNM 10X 5h	8.47 × 10^6^ ± 3.88 × 10^6^	~0	~0
TNM 10X 15h	1.15 × 10^7^ ± 2.19 × 10^6^	~0	~0
TNM 10X 24h	8.85 × 10^7^ ± 4.69 × 10^6^	~0	~0
TNM 0.1X 2h	1.36 × 10^7^ ± 1.51 × 10^6^	~0	~0
TNM 0.1X 5h	3.18 × 10^7^ ± 1.39 × 10^7^	~0	~0
TNM 0.1X 15h	1.05 × 10^7^ ± 1.22 × 10^6^	~0	~0
TNM 0.1X 24h	8.13 × 10^7^ ± 4.69 × 10^6^	~0	~0

## Data Availability

Data will be made available upon request.
